# Genome mining of *Streptomyces bambergiensis* AC-800 unravels the biosynthetic gene cluster for inhibitors of prolyl hydroxylase fibrostatins

**DOI:** 10.1038/s41598-025-17585-y

**Published:** 2025-09-01

**Authors:** Jaime Felipe Guerrero Garzón, Martin Zehl, Olha Schneider, Inmaculada Tocino Marquez, Christian Rückert-Reed, Jörn Kalinowski, Sergey B. Zotchev

**Affiliations:** 1 Department of Pharmaceutical Sciences, Division of Pharmacognosy, University of Vienna, Vienna, 1090 Austria; 2 Department of Analytical Chemistry, Faculty of Chemistry, University of Vienna, Vienna, 1090 Austria; 3 Medical School OWL, Bielefeld University, 33615 Bielefeld, Germany; 4 Technology Platform Genomics, Center for Biotechnology (CeBiTec), Bielefeld University, 33615 Bielefeld, Germany

**Keywords:** *Streptomyces bambergiensis*, Genome, Biosynthetic gene clusters, Secondary metabolites, Fibrostatin, Biotechnology, Drug discovery

## Abstract

*Streptomyces bambergiensis* AC-800 is known as a producer of moenomycin family antibiotics active against gram-positive bacteria. Complete genome sequencing of *S. bambergiensis* revealed 3 replicons represented by the linear chromosome (7,652,101 bp) and two linear plasmids, pSB1 (418,507 bp) and pSB2 (81,486 bp). Analysis of the chromosome for the presence of secondary metabolite biosynthesis gene clusters (BGCs) revealed 25 BGCs, while other 4 were located on the linear plasmid pSB1. The bioinformatics-based analysis of the moenomycin BGC provided new insights into its biosynthesis. The largest reported polyketide synthase gene cluster spanning over 190 kb was identified on the pSB1 plasmid, with its putative product likely to be represented by a 67-membered glycosylated macrolide related to stambomycins. Co-cultivation of *S. bambergiensis* AC-800 with a strain of *Rhodococcus* isolated from a fresh-water bryozoan induced production of a red pigment tentatively identified as fibrostatin. CRISPR-BEST-assisted inactivation of the only PKSIII-encoding gene abolished the production of fibrostatin, allowing the identification of the previously unreported fibrostatin BGC. Subsequent secondary metabolomics of *S. bambergiensis* cultivated in different media revealed production of both known and presumably novel compounds. This study sets a stage for further investigation of this strain by means of genome mining that may result in the discovery of novel bioactive natural products.

**Supplementary Information:**

The online version contains supplementary material available at 10.1038/s41598-025-17585-y.

## Introduction

Gram-positive filamentous bacteria of the genus *Streptomyces* are known as producers of a wide variety of biologically active secondary metabolites (SMs). Many of such SMs display anti-microbial and cytotoxic activities, and some have been developed into antibiotics and antitumor agents (e.g. tetracycline, amphotericin B, doxorubicin, mytomycin etc.). Each SM is the result of a series of reactions catalyzed by enzymes encoded by genes present in a particular biosynthetic pathway, and thus its production has certain costs for the producing cells in terms of precursors and energy. This can be taxing for the bacteria, and it is thus not surprising that production of SMs is limited to situations where a survival benefit can be expected. It is also logical that genes encoding enzymes involved in SM biosynthesis are clustered in the bacterial genomes to allow coordinated expression and regulation. Recent sequencing of *Streptomyces* genomes revealed many BGCs (20–50 per genome), some of which may govern production of novel bioactive compounds, not detected previously in a conventional screening^1^. In most cases, these BGCs are not expressed under laboratory conditions, and need to be activated to achieve production of the corresponding compounds. Therefore, the genomes of *Streptomyces* represent a potential treasure trove in terms of drug discovery. A number of techniques have recently been developed to address this issue, including co-cultivation with other microorganisms^2^addition of small molecules acting as activators of secondary metabolism^3^ ribosome engineering^4^ manipulation of pathway-specific regulators, and heterologous expression of SM biosynthesis gene clusters^5^. All these approaches can be applied to a single bacterial strain to implement the One-Strain-Many-Compounds (OSMAC) strategy^6^. The latter strategy can be implemented via simply changing the cultivation conditions, e.g. nutrients, cultivation time and temperature, co-cultivation with other organisms etc. However, having a high-quality genome sequence of a bacterial SM producer and being able to genetically manipulate it brings about new opportunities for implementation of the OSMAC strategy via targeting specific BGC of interest.

In this work, we investigated *Streptomyces bambergiensis* AC-800, formerly known as *Streptomyces bambergiensis* S800, which harbors two linear plasmids^7^. Along with several other *Streptomyces*, such as *Streptomyces ghanaensis* ATCC 14,672, *S. ederensis* ATCC 15,304 and *S. geysiriensis* ATCC 15,303, it produces a complex of glycolipid antibiotics known as moenomycins or flavomycins^8^. While no other SMs have been described so far for this strain, efficient gene transfer methods have been reported for *S. bambergiensis* AC-800^9^, making it suitable for genetic engineering strategies and OSMAC approach. Here, we report the complete sequence of the *S. bambergiensis* AC-800 genome, its analysis for special genome features and SM biosynthesis gene clusters, activation of a cryptic fibrostatin BGC via co-cultivation, and first insights into the secondary metabolome of this strain.

## Results and discussion

### Sequence of the *Streptomyces bambergiensis* AC-800 genome

Complete genome assembly revealed three replicons, one represented by a linear chromosome of 7,652,101 nt, and two by linear plasmids designated pSB1 (418,507 nt) and pSB2 (81,486 nt). In addition to the NCBI Prokaryotic Genome Annotation Pipeline (PGAP)^10,11^ the three replicons were annotated at the Joint Genome Institute (JGI) using the Integrated Microbial Genomics platform, and the genome statistics are presented in Table 1. The linear plasmids pSB1 and pSB2 were detected earlier in *S. bambergiensis* S800 (older name for AC-800), a strain presumed to be identical to ATCC 13,879, using pulsed field gel electrophoresis (PFGE)^7^. Although the reported sizes of the plasmids were quite different (350 kb and 50 kb), this most likely reflects inaccuracy of size estimation based on the PGFE markers available back then. It is noteworthy that the JGI annotation pipeline predicted that only 0.45% of the chromosomally located genes seem to have been acquired via horizontal gene transfer (HGT), while percentages of HGT-derived genes for the plasmids pSB1 and pSB2 were 39% and 59%, respectively. Both plasmids harbored genes encoding the machinery for conjugative DNA transfer. Taking together, these facts strongly suggest that linear plasmids in streptomycetes serve as shuttles that can acquire foreign genes and shuttle them between species.

**Table 1:** Genome features of *S. bambergiensis* AC-800.

Attribute	Chromosome	pSB1	pSB2	% of total
Genome size (bp)	7,652,101	418,507	81,486	100.00
DNA coding region (bp)	6,488,14	385,618	62,536	
DNA G + C content (bp)	5,511,001	309,341	56,379	
DNA scaffolds/contigs	1/1	1/1	1/1	
Total genes	6,711	171	89	100.00
Protein-coding genes	6,619	171	89	
Pseudo genes	222	14	4	
RNA genes	91	0	0	
rRNA genes	18	0	0	
tRNA genes	71	0	0	
Genes with function prediction (protein)	5742	148	39	

As all linear replicons reported up to date, the chromosome and the plasmids of *S. bambergiensis* featured terminal inverted repeats (TIRs). In particular, the chromosomal TIRs spanned 2.28 kb with 100% identical sequences, while TIRs of pSB1 and pSB2 plasmids spanned 2.28 kb (100% identity) and 0.3 kb (imperfect, 85% identity), respectively. We compared the ends of the *S. bambergiensis* AC-800 chromosome and those of the pSB1 plasmid and determined that they share 7.84 kb 100% identical sequences, including the 2.28 kb TIRs, which might be due to recombination-driven exchange. These terminal DNA fragments encoded putative DEAD/DEAH box helicase and DDE-type integrase/transposase/recombinase, suggesting the importance of terminal region for both replication and recombination, the latter presumably being important for acquisition of new DNA fragments into the replicons. Notably, *S. bambergiensis* ATCC 13,879, the presumed parental strain for AC-800, was re-classified to *Streptomyces prasinus* ATCC 13,879 in 2016 based on multi-locus sequence analysis^12^. The *S. prasinus* ATCC 13,879 genome deposited in GenBank (ASM870444v1) consists of 1 chromosome of 7,647,592 nt, which is thus 4,509 nt smaller than the chromosome of AC-800. Also, no linear plasmids have been reported for *S. prasinus* ATCC 13,879. Comparison of the chromosomes of AC-800 and ATCC 13,879 using ANI Calculator^13^ yielded an OrthoANIu value of 99.97%, which clearly indicates that the two strains are almost identical, at least at the level of their chromosomes. We were unable to identify any TIRs at the ends of *S. prasinus* ATCC 13,879 chromosome, which is very unusual for streptomycetes, and may suggest that the chromosomal ends were not completely sequenced.

### Secondary metabolite biosynthesis gene clusters of *S. bambergiensis* AC-800

**Fig. 1:**
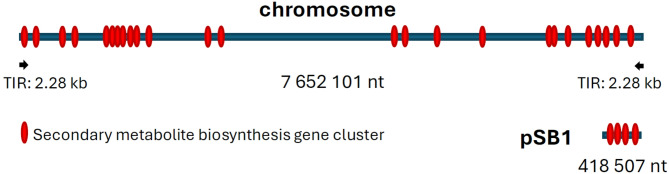
Localization of the secondary metabolite biosynthesis gene clusters on the chromosome and pSB1 plasmid of *S. bambergiensis* AC-800.

In order to reveal the SM biosynthesis potential of *S. bambergiensis*, all three replicons were scanned for the presence of BGCs using the online software tool antiSMASH 7.0^14^. This analysis was followed by a manual inspection of the identified gene clusters using BLAST online tools, which helped to detect homologous gene clusters in the genomes of other bacteria. In total, 29 BGCs were identified – 25 of them localized to the chromosome, and 4 to the pSB1 giant linear plasmid (Fig. 1). The full list of the gene clusters is given in Table 2, along with putative or defined products and homologous clusters detected in other streptomycetes excluding *S. prasinus* ATCC 13,879, which chromosome had almost the same BGCs as AC-800. The main differences between the ATCC and AC-800 strains chromosomes in this respect are in BGCs 20 and 21 (Table 2). BGCs that are marked with # in Table 2 appear to be present, at the time of analysis, only in *S. prasinus* and *S. bambergiensis*, while other BGCs can be found in both these strains and also in other streptomycetes Lanthipeptide-specifying BGC20, although highly similar in gene content and arrangement in both strains, differs in the genes encoding the pre-peptides, while BGC21 for a hybrid NRPS-PKSI system has identical genes but organized in a different way.

**Table 2:** Secondary metabolite biosynthesis gene clusters identified in the genome of *Streptomyces bambergiensis* AC-800 using antismash 7.0 software followed by manual curation.

No	Cluster type (antiSMASH)	Presence in another bacterium	Putative product
1	NRPS-PKS type I	*-* ^*#*^	Hybrid NR peptide-polyketide
2	Terpene	*Streptomyces* sp. KMM 9044	Terpenoid
3	NRPS	*-* ^*#*^	NR decapeptide
4	Terpene	All *Streptomyces*	**Hopanoids**
5	NRPS-PKS type I	*-* ^*#*^	Hybrid NRS peptide-polyketide
6	PKS type III	*Streptomyces* sp. H-KF8	**Fibrostatin**
7	Terpene	*-* ^*#*^	Isorenieratene-like terpenoid
8	Siderophore	*Streptomyces* sp. H-KF8	Siderophore
9	Terpene	All *Streptomyces*	**Geosmin**
10	RiPP, NRPS-PKS type I	*Streptomyces rubrolavendulae* MJM4426	Hybrid NR peptide-polyketide, trans-AT
11	Terpene	*Streptomyces viridosporus* T7A	**Albaflavenone**
12	NRPS-PKS type I	*Streptomyces prasinopilosus* CGMCC 4.3	Polycyclic tetramate macrolactam
13	Siderophore	*Streptomyces prasinopilosus* CGMCC 4.3	**Desferrioxamine**,** terragines**
14	Melanin	*Streptomyces prasinopilosus* CGMCC 4.3	**Melanin**
15	PKS type I and II	*Streptomyces* sp. TAA040	Polyketide
16	Redox cofactor	*Streptomyces* sp. NRRL F-525	Redox cofactor
17	Ectoine	*Streptomyces prasinopilosus* CGMCC 4.3	**Ectoine**
18	PKS type II	*Streptomyces prasinopilosus* CGMCC 4.3	**Spore pigment**
19	NRPS	*-* ^*#*^	NR heptapeptide
20	Lanthipeptide	*-*	FxLD family lanthipeptides
21	NRPS-PKS type I	*Streptomyces prasinopilosus* CGMCC 4.3	Hybrid NR peptide-polyketide
22	Lasso peptide	*Streptomyces prasinopilosus* CGMCC 4.3	Lasso peptide class II
23	NRPS	*-* ^*#*^	NR peptide
24	Phosphoglycolipid-nucleoside	*Streptomyces ghanaensis* ATCC 14,672	**Moenomycins**,** nosokomycins**
25	NRPS	*-* ^*#*^	NR 13-aa peptide
26*	PKS type I	*-*	Stambomycin-like macrolide
27*	PKS type I-NRPS	*Streptomyces durmitorensis* DSM41863	Hybrid NRS peptide-polyketide
28*	Butyrolactone	*Streptomyces* sp. H-KF8	Butyrolactone
29*	NRPS	*Streptomyces* sp. MAR25Y5	Putative lipopeptide

NR – non-ribosomally synthesized. Shaded cells highlight potentially unique gene clusters, known metabolites are shown in bold font.

*Gene clusters located on the giant linear plasmid pSB1.

^#^Present in *S. prasinus* ATCC 13,879, but not in other streptomycetes (data from 15.01.2025).

Nine of the gene clusters located on the *S. bambergiensis* chromosome presumably specify biosynthesis of some known metabolites: hopanoids, albaflavenone, isorenieratene, geosmin, ectoine, moenomycin, spore pigment, desferroxamine, and melanin. Four chromosomally located gene clusters and one located on pSB1 appeared to be unique, as their homologues could not be detected in the bacterial genomes available in public databases. The rest of the gene clusters had close homologues in the genomes of various *Streptomyces* sp. (Table 2).

### Insights into the evolution of the giant PKSI gene cluster

**Fig. 2:**
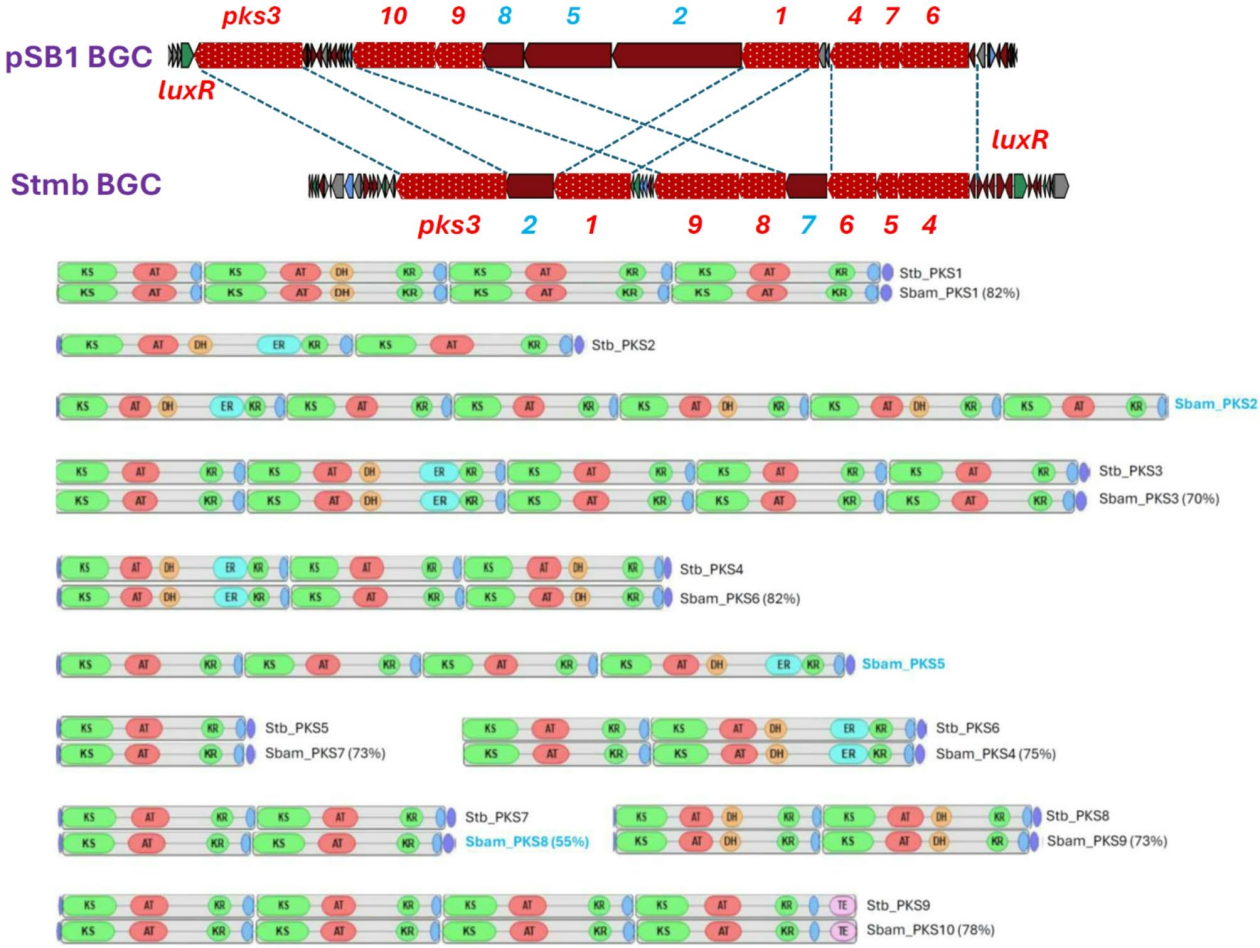
PKS type I gene cluster located on the giant linear plasmid pSB1: evolutionary relations with the stambomycin gene cluster of *S. ambofaciens*. PKS genes are numbered according to the biosynthetic order predicted for pSB1 PKS BGC by antiSMASH and experimentally verified for stambomycins. Homologous PKS genes are shaded and marked with dotted lines and those designated in blue font are unique for each cluster. Stb _PKS – PKSI of the stambomycin BGC; Sbam_PKS – PKSI of the pSB1-licated BGC in *S. bambergiensis*.

**Fig. 3:**
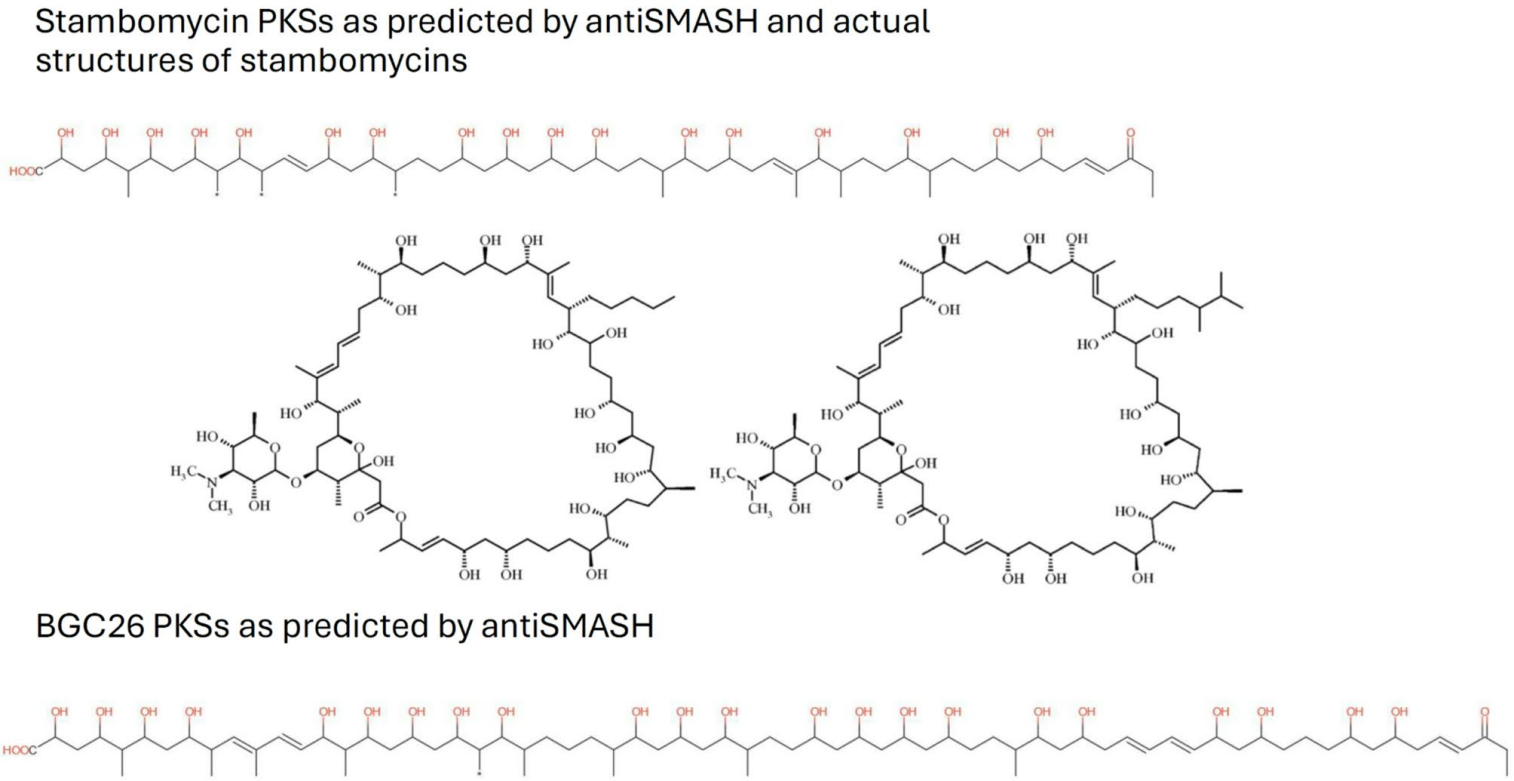
Putative linear polyketide products of PKS systems encoded by stambomycin BGC and BGC26 of *S. bambergiensis* AC-800, as predicted by antiSMASH 7.0 and actual structures of stambomycins reported by Laureti et al.^19^.

Inspection of the PKSI gene cluster 26 encoded by the linear plasmid pSB1 revealed that it most likely specifies biosynthesis of a glycosylated macrocyclic lactone related to stambomycins. The latter SMs were produced by *Streptomyces ambofaciens* ATCC 23,877 only upon overexpression of a pathway-specific regulator of the LuxR type^19^. Examination of the PKS modules encoded by cluster 26 suggested that, in contrast to stambomycins, which feature a 51-membered macrolactone ring^19^ a putative product of this cluster may possess an unprecedented 67-membered ring. Comparison of the BGC26 PKS genes and module/domain organization of the corresponding proteins with those from the stambomycin BGC revealed a clear evolutionary relatedness between these clusters (Fig. 2). First, we performed alignments of the PKS proteins encoded by 10 genes in *S. bambergiensis* BGC26 with potential counterparts encoded by 9 genes in the stambomycin BGC. This analysis revealed that 7 out of 10 PKS proteins encoded by BGC26 were homologous to 7 PKS proteins from the stambomycin cluster and had same module/domain composition (Fig. 2). Further antiSMASH-assisted analysis of the BGC26 PKSs focusing on the docking domains allowed to suggest the order, in which the PKS proteins would synthesize the polyketide chain, reflected by numbers over the PKS genes in Fig. 3. At the same time, we analyzed intergenic regions between seemingly co-transcribed PKS genes using the promoter-searching software SoftBerry^20^. This result suggested that PKS genes 6, 7 and 4 in BGC26 are likely to be transcriptionally coupled and thus their products are likely to act successively, as their stambomycin PKSs counterparts 4, 5 and 6. We cannot resolve this discrepancy without experimental evidence, especially because we were not able to detect the product of BGC26 even after overexpression of LuxR gene from this cluster *in S. bambergiensis* (data not shown). Hence, the antiSMASH prediction of a polyketide chain resulting from the action of BGC26 PKSs remains speculative as to the arrangement of the building blocks. According to the antiSMASH-based prediction of building blocks to be used for assembly of BGC26-encoded PKS proteins, they would use 1 propionyl-CoA, 9 methylmalonyl CoA and 24 malonyl-CoA to generate a polyketide chain shown in Fig. 3. When cyclized, a 67-membered macrolactone ring will likely to be formed, which would feature 9 methyl and 22 hydroxy (if no hemiketal is formed) groups. This would contrast stambomycin molecule with 8 methyl and 17 hydroxy groups predicted by antiSMASH, but also true according to the actual chemical structure of stambomycins^19^. We next compared the composition of genes other than those coding for PKS in the stambomycin cluster and BGC26, verifying potential homologues using alignment of amino acid sequences of their products. The results of this analysis, presented in Table 3, suggest that the putative 67-membered macrolactone ring synthesized by BGC26 PKSs is most likely glycosylated with at least one sugar moiety (presumably an aminosugar), and is also modified by two P450 cytochromes in a manner similar to those in stambomycin biosynthesis. One of such reactions is likely to be hydroxylation shown to be required for macrolactonization in stambomycin biosynthesis^21^. BGC26 does not have many gene homologues present in the stambomycin BGC, while containing several genes not found in the latter (Table 3). While it speculation on the function of these genes in the biosynthesis of BGC26-specified compound seems unreasonable, it should be noted that ABC transporters encoded by stambomycin BGC and BGC26 are very dissimilar, indirectly supporting the idea that products of these two clusters are quite dissimilar.

**Table 3:** Comparative analysis of genes in the stambomycin BGC and BGC26 of *S. bambergiensis* AC-800.

Stambomycin BGC	pSB1 PKS BGC	Putative function of encoded protein	Identity on amino acid level, %
SAM23877_7104	-	Glucose-1-phosphate thymidylyltransferase	-
SAM23877_7105	-	dTDP-glucose 4,6-dehydratase	-
SAM23877_7106	SBAM_006731	Type II thioesterase	56
SAM23877_7107	SBAM_006728	Large ATP-binding regulator of the LuxR family	61
SAM23877_7108	-	Carboxyl transferase	-
SAM23877_7109	-	Acyl-CoA synthetase	-
SAM23877_7110	SBAM_006736	Glycosyltransferase	82
SAM23877_7111	SBAM_006733	Aminotransferase (D-mycaminose biosynthesis? )	83
SAM23877_7112	SBAM_006732	Cytochrome P450	86
SAM23877_7113	SBAM_006752	Cytochrome P450	88
SAM23877_7114	SBAM_006751	Type I polyketide synthase	82
SAM23877_7115	SBAM_006750	Type I polyketide synthase	73
SAM23877_7116	SBAM_006749	Type I polyketide synthase	75
SAM23877_7117	-	Type I polyketide synthase	-
SAM23877_7118	SBAM_006742	Type I polyketide synthase	73
SAM23877_7119	SBAM_006741	Type I polyketide synthase	78
SAM23877_7120	-	3,4 keto-isomerase	-
SAM23877_7121	-	N-dimethyltransferase	-
SAM23877_7122	-	ABC transporter, ATP-binding subunit	-
SAM23877_7123	-	ABC transporter permease	-
SAM23877_7124	-	Two-component system sensor kinase	-
SAM23877_7125	-	Two-component system response regulator	-
SAM23877_7126	SBAM_006746	Type I polyketide synthase	82
SAM23877_7127	-	Type I polyketide synthase	-
SAM23877_7128	SBAM_006745	Type I polyketide synthase	70
SAM23877_7129	-	Endoribonuclease	-
SAM23877_7130	-	Transcriptional regulator (DeoR)	-
SAM23877_7131	-	Acetyltransferase	-
SAM23877_7132	-	GntR family transcriptional regulator	-
SAM23877_7133	-	Amidohydrolase	-
SAM23877_7134	-	D-threo-aldose 1-dehydrogenase	-
SAM23877_7135	-	2-(S)-hydroxypropyl-CoM dehydrogenase	-
SAM23877_7136		L-fuconate dehydratase	
Genes within the predicted borders of BGC26 not present in the stambomycin BGC			
	SBAM_006730	Crotonyl-CoA carboxylase/reductase	
	SBAM_006731	Alpha/beta fold hydrolase	
	SBAM_006734	NDP-hexose 2,3-dehydratase	
	SBAM_006735	Class I SAM-dependent methyltransferase	
	SBAM_006737	NAD-dependent epimerase/dehydratase	
	SBAM_006738	dTDP-4-dehydrorhamnose 3,5-epimerase	
	SBAM_006739	ABC transporter permease	
	SBAM_006740	ABC transporter ATP-binding protein	
	SBAM_006742	SDR family NAD(P)-dependent oxidoreductase	
	SBAM_006747	Hypothetical protein	
	SBAM_006748	ABC transporter ATP-binding protein	
	SBAM_006749	SDR family NAD(P)-dependent oxidoreductase	
	SBAM_006753	lysyl oxidase family protein	
	SBAM_006754	MarR family transcriptional regulator	
	SBAM_006755	MFS transporter	
	SBAM_006756	4’-phosphopantetheinyl transferase	
	SBAM_006757	Beta-ketoacyl synthase with LINKS motif	
	SBAM_006758	Acyl carrier protein	

### Secondary metabolites produced by *S. bambergiensis* AC-800 and their cognate biosynthetic gene clusters

The extracts of *S. bambergiensis* AC-800 grown in four different media (5288, PM4-1, NIIGEN, and A-3 M) were analyzed by LC-MS to tentatively identify secondary metabolites produced by this strain (Table 4). Moenomycin-related compounds were detected in low concentrations in all media except A-3 M. In 5288, moenomycin A and nosokomycin B were the predominant congeners (Figures S1 and S2), while in NIIGEN and PM4-1 moenomycin A_12_ was most abundant (Figure S3)^8,22^. Desferrioxamine B and several related hydroxamate sideropohores (Figures S4-S8) as well as the γ-aminobutyrate-derived ureas gaburedins A-D (Figures S9-S12) were found in higher amounts. These latter two secondary metabolite families are very commonly produced by *Streptomyces* sp^23,24^. Notably, we could not identify genes encoding GbnA and GbnB proteins reported to be required for the biosynthesis of gaburedins. It is thus plausible that these g-aminobutirate ureas are synthesized via an alternative, as yet undiscovered pathway. Furthermore, two polycyclic tetramate macrolactams, presumably products of BGC 12, were detected (Figures S13 and S14). One of them was matched by GNPS to alteramide A, but there are too many structurally similar isomers described for both congeners to safely identify them solely based on LC-MS data.

**Table 4:** Secondary metabolites tentatively identified by LC-MS in *Streptomyces bambergiensis* AC-800 cultures in at least one of four different media (5288, PM4-1, NIIGEN, and A-3 M).

No	*R*_t_ [min]	m/z [M + H]^+^	m/z [M + 2 H]^2+^	Sum formula	m/z calcd.	Δm/z [ppm]	Tentative ID	BGC	The natural products atlas
1	4.0	247.1287		C_10_H_18_N_2_O_5_	247.1288	0.5	Gaburedin D	28	*NPA028620*
2	6.1	261.1443		C_11_H_20_N_2_O_5_	261.1445	0.8	Gaburedin C	28	*NPA028619*
3	6.5	261.1442		C_11_H_20_N_2_O_5_	261.1445	1.1	Gaburedin B	28	*NPA028618*
4	6.8	361.2446		C_16_H_32_N_4_O_5_	361.2445	-0.1	CAS: 252325-60-3	13	
5	7.4	295.1287		C_14_H_18_N_2_O_5_	295.1288	0.5	Gaburedin A	28	*NPA028617*
6	9.0	547.3449	274.1762	C_24_H_46_N_6_O_8_	547.3450	0.2	CAS: 1884272-02-9	13	
7	9.4	561.3606	281.1840	C_25_H_48_N_6_O_8_	561.3606	0.0	Desferrioxamine B	13	*NPA009012*
8	11.9	443.2499		C_20_H_34_N_4_O_7_	443.2500	0.2	Terragine F (CAS: 2779605-10-4)	13	
9	12.1	603.3712	302.1893	C_27_H_50_N_6_O_9_	603.3712	0.1	Desferrioxamine D1 (CAS: 5722-48-5)	13	
10	17.7	410.0905		C_18_H_19_NO_8_S	410.0904	-0.2	Fibrostatin C/D/E	6	*NPA020756*
11	23.2	511.2802		C_29_H_38_N_2_O_6_	511.2803	0.1	Polycyclic tetramate macrolactam	12	
12	24.7	495.2851		C_29_H_38_N_2_O_5_	495.2853	0.4	Polycyclic tetramate macrolactam	12	
13	26.3	1486.6453	743.8268	C_64_H_104_N_5_O_32_P	743.8274	0.8	Nosokomycin B	24	*NPA006746*
14	26.8	1568.6525	784.8297	C_68_H_106_N_5_O_34_P	784.8301	0.6	Moenomycin A_12_	24	*NPA001493*
15	26.9	1582.6674	791.8375	C_69_H_108_N_5_O_34_P	791.8379	0.5	Moenomycin A	24	*NPA007626*

### Activation of fibrostatin biosynthesis via co-cultivation and identification of its biosynthetic gene cluster

Despite using four different fermentation media, we were able to detect the products of only 5 BGCs in the cultures of *S. bambergiensis* AC-800 by a standard dereplication approach. Co-cultivation of actinomycetes with mycolic acid-containing bacteria (MACB) is a known approach to activate BGCs which are either cryptic or silent^25,26^. Thus, in an attempt to activate production of additional secondary metabolites, *S. bambergiensis* AC-800 was co-cultivated with a new MACB isolate from the freshwater bryozoan *Cristatella mucedo*, *Rhodococcus* sp. CML ISP2-1-52 in A-3 M medium. This co-cultivation triggered production of compound(s) that gave the culture a reddish color absent in the respective monocultures. Comparative LC-MS analysis led to the tentative identification of these compounds as fibrostatin C (Figure S15), or alternatively one of the isomers fibrostatin D or E, which are *N*-acetyl-L-cysteinyl-containing 1,4-naphthoquinone derivatives first isolated from *Streptomyces catenulae*^27^. Fibrostatin C was shown to inhibit prolyl hydroxylase purified from chicken embryo and type I collagen biosynthesis in the uterus of the immature rat^28^. The chemical structure of the compounds suggests that they are biosynthesized by a type III polyketide synthase (PKSIII), leaving BGC 6 the only plausible candidate in the *S. bambergiensis* AC-800 genome (Table 2). To validate whether BGC6 is governing fibrostatin biosynthesis, a PKSIII gene knockout mutant was generated. A stop codon was introduced instead of codon W117 in the gene *SBAM_000974* encoding the PKSIII enzyme using pCRISPR-cBEST technology^29^. This mutation results in early termination of *SBAM_000974* translation and generation of a truncated enzyme. The production of fibrostatin C was completely abolished in the *SBAM_000974* knockout (KO) mutant of *S. bambergiensis* AC-800 upon co-cultivation with *Rhodococcus* sp. CML ISP2-1-52, thus proofing that BGC6 is indeed responsible for the fibrostatin biosynthesis (Figure S16). We also observed the disappearance of the reddish color in such co-cultures. We also detected very low amounts of fibrostatin C in the *S. bambergiensis* AC-800 wild type monocultures grown in 5288 and PM4-1 media, but not in the KO strain under the same conditions (Figure S17).

**Fig. 4:**
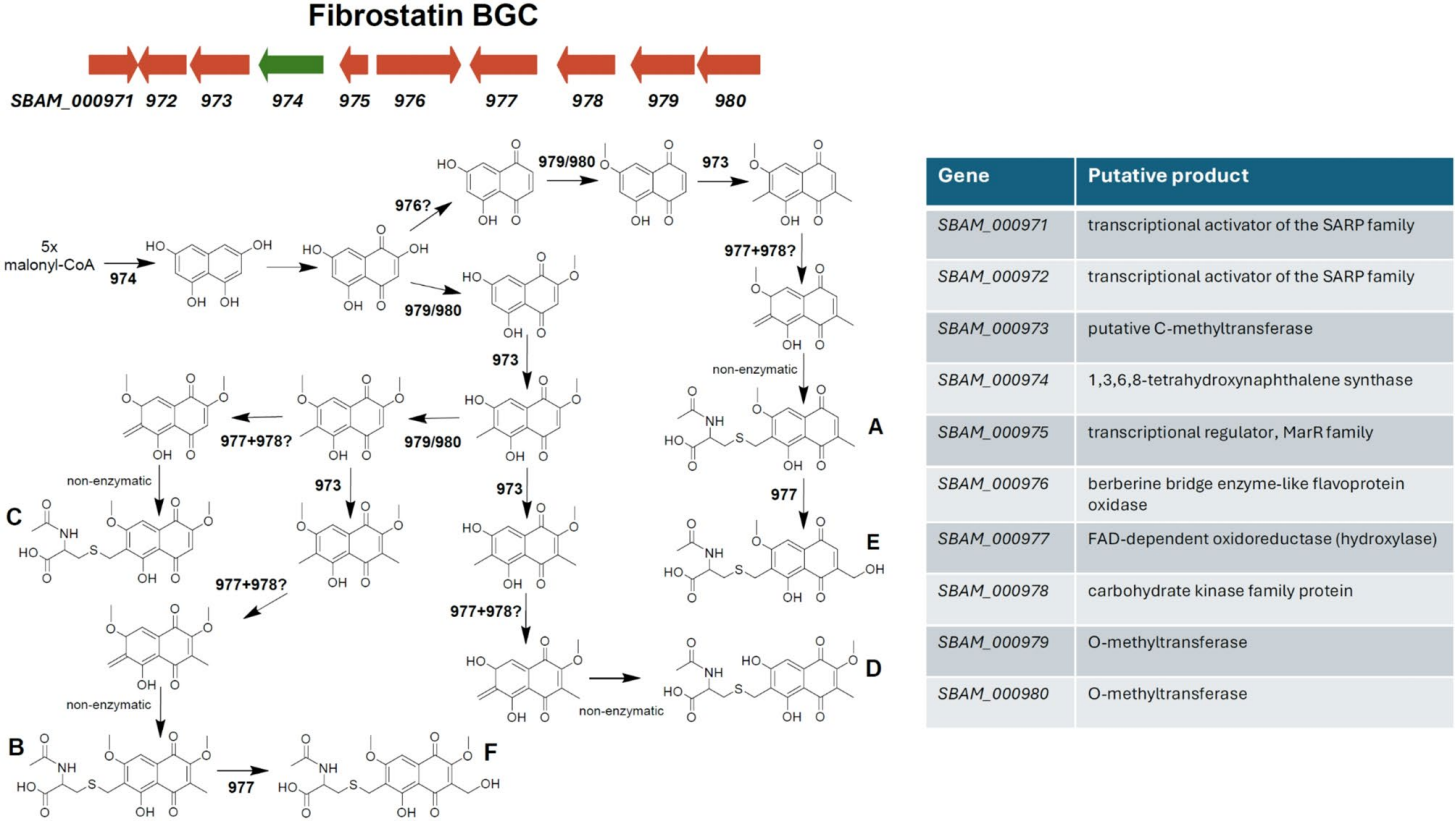
Proposed fibrostatin biosynthesis pathway in *S. bambergiensis* AC-800.

Besides the PKSIII-coding gene, the fibrostatin BGC contains genes encoding two O-methyltransferases, several oxidoreductases and a P450 monooxygenase, which could play specific roles in the proposed fibrostatin biosynthesis pathway (Fig. 4). However, BGC6 does not contain any gene which product could be responsible for the installation of the *N*-acetyl-L-cysteinyl (NAC) group. It thus seems plausible that fibrostatin C and other naphthoquinone NAC adducts are produced non-enzymatically by conjugation of NAC or mycothiol to a reactive naphthoquinone product of BGC6, as described for several naphthoquinones^30^. The red color observed in the cultures might be mainly explained by these poorly soluble fibrostatin congeners not efficiently extracted, while fibrostatin C is detected as main congener due to its higher solubility in methanol. This hypothesis is supported by the detection of several low intensity peaks that have LC-MS and UV/Vis data fitting to non-conjugated naphthoquinones, which could however not be unequivocally identified (data not shown).

Following a thorough analysis of the DNA regions surrounding the PKSIII-encoding gene we suggest a fibrostatins biosynthetic gene cluster encompassing 10 genes. Based on the proposed functions of the gene products we hypothesize on the possible biosynthetic pathway for fibrostatins A-F, as presented in Fig. 4. The biosynthesis starts with condensation of five malonyl-CoA units by PKSIII to form 1,3,6,8-tetrahydroxynaphthalene, which is spontaneously oxidized to flaviolin. At this point, the pathway must diverge into two branches, one leading to the biosynthesis of fibrostatins A and E, and another yielding congeners B, C, D, and F. The pathway to A and E congeners must include a C2-dehydroxylation step, which may be catalyzed by the berberine bridge enzyme-like FAD-dependent oxidase SBAM_000976. This enzyme family, termed BBE-like oxidases, catalyze a wide range of reactions, typically resulting in complex substrate modifications and rearrangements^31^. The next steps toward fibrostatins A and E would be the addition of one *O*- and two *C*-methyl groups followed by the formation of an *ortho*-quinone methide (ο-QM). The latter could be achived by hydroxlation of the methyl group on C6 by the FAD-dependent hydroxylase SBAM_000977 followed by phosphorylation and subsequent phosphate elimination involving kinase SBAM_000978. This mechanism may be similar to that described for YcaO-like enzymes that catalyze phosphorylation of a peptide backbone amide followed by deprotonation and phosphate elimination to yield azoline heterocycles^32^. Alternatively, direct oxidation of the ring system harboring the *C*-methyl group to the ο-QM could be catalyzed by the BBE-like oxidase. Similar reactions involving ο-QM intermediate formation by BBE-like oxidase were proposes in the biosynthsis of several natural products, including e.g. chlorizidine A from *Streptomyces* sp. CNH-287^31^. The ο-QM represents an unstable intermediate that can easily react with thiols^33^such as *N*-acetyl cysteine, thus leading to the formation of fibrostatin A. Subsequent hydroxylation of the methyl group on C3 by the FAD-dependent hydroxylase SBAM_000977 would then yield fibrostatin E. The formation of fibrostatins B, C, and F presumably follows the same logic, where, however, the C2-dehydroxylation is blocked by *O*-methylation (Fig. 4).

## Materials and methods

### Culture conditions

*E. coli* DH5alpha and commercial *E. coli* Mach1 T1 were used to maintain plasmids. *E. coli* ET12567 (pUZ8002) was used to perform conjugations of plasmids into *S. bambergiensis* AC-800. All *E. coli* strains were cultivated in liquid and solid LB-medium at 37 °C. *Streptomyces* sp. were grown at 30 °C in soy flour medium (SFM)^34^ followed by preparation of spore suspensions in 20% glycerol. Appropriate antibiotics were supplemented as necessary (50 µg/mL apramycin; 50 µg/mL nalidixic acid; 25 µg/mL kanamycin; and 25 µg/mL chloramphenicol). *Rhodococcus* sp. CML ISP2-1-52 was isolated from a colony of the fresh-water bryozoan *Cristatella mucedo* after colony homogenization and plating of serial dilutions on the ISP2 magar medium.

### DNA isolation, genome sequencing, assembly, and annotation

Genomic DNA was extracted using the NucleoSpin Microbial DNA Mini kit (Macherey & Nagel, Germany) according to the manufacturer’s instructions. From this gDNA, three kits (Illumina, all used according to the manufacturer’s instructions) were used to create three different sequencing libraries: the Nextera XT DNA Library Kit, the TruSeq DNA PCR Free Library Kit (due to high variation in coverage of the Nextera XT DNA library), and the Nextera Mate Pair Library Preparation Kit. For the Mate Pair library, the input DNA was sheared to a size of approximately 8 kbp using a g-Tube (Covaris). The libraries were sequenced on a MiSeq sequencer (Illumina) using the either MiSeq Sequencing Kit v2 in a 2 × 250 nt run for the Nextera XT DNA library or the MiSeq Sequencing Kit v3 in 2 × 300 nt runs for the TruSeq DNA PCR Free and Nextera Mate Pair libraries. The paired reads from the Nextera XT and TruSeq libraries were then trimmed using Trimmomatic 0.36^35^ (http://www.usadellab.org/cms/index.php?page=trimmomatic) in PE mode with trimmers ILLUMINACLIP: [NexteraPE-PE.fa| TruSeq3-PE-2.fa]:2:30:10, SLIDINGWINDOW:4:15, and MINLEN:25. The paired reads of the Mate Pair library were merged using FLASH^36^ (https://ccb.jhu.edu/software/FLASH/) with a maximum overlap (-M) of 250 and then split and oriented using Cutadapt 5.1^37^ (https://cutadapt.readthedocs.io/en/stable/) and SeqTK 1.5^38^ (https://github.com/lh3/seqtk). The data of all three libraries was then assembled using Newbler (454 Life Sciences), resulting in an initial assembly of 161 in 8 scaffolds. Using consed 19^39^ (https://bioweb.pasteur.fr/packages/pack@consed@19), these could be manually assembled into three contigs, one of which each represented the complete sequence of the three linear replicons: the chromosome and the plasmids pSB1 and pSB2. The genome was then annotated using PGAP 4.0^40,41^ (https://github.com/ncbi/pgap).

### Gene inactivation using crispr/cbest system

CRISPR-cBEST system was used to inactivate the *SBAM_000974* gene in *S. bambergiensis* AC-800. The identification of protospacers compatible with CRISPR-cBEST was done using CRISPy-web 2^42^ (https://crispy.secondarymetabolites.org/#/input) (Table S1). The protospacers were cloned into the linearized pCRISPR-cBEST plasmid as specified previously^29^. The resulting constructs were individually introduced in *S. bambergiensis* AC-800 via conjugation from *E. coli* ET12567/pUZ8002^34^. Apramycin (50 µg/mL) was used for selection of recombinant *Streptomyces* strains. Primers that amplifyseveral-hundred base pairs fragment containing the edited codon were designed (Table S2). Colony PCR was used to amplify the designed DNA fragments directly from the *S. bambergiensis* AC-800 colonies. Lastly, the PCR products were cleaned up by Monarch^(R)^ PCR and DNA Cleanup Kit (New England Biolabs^(R)^ Inc.) and then sequenced at Microsynth, Austria. pCRISPR-cBEST plasmid was curated from KO strain after 24 h cultivation at 37 °C and 200 rpm in TSB medium.

### Fermentation conditions, extraction, and analysis of secondary metabolites

*S. bambergiensis* AC-800 was fermented in medium 5288 (soy flour 10 g, glycerol 15 g, NaCl 5 g, CaCO_3_ 1 g, CoCl_2_·7H_2_O 1 mg, in 1 L of distilled water), NIIGEN (soy flour 30 g, corn steep liquor 30 g, starch 30 g, CaCO₃ 4 g, (NH₄)₂SO₄ 3 g, K₂HPO₄ 1 g, NaCl 2 g, in 1 L of distilled water), PM4-1 (glucose 15 g, soy flour 15 g, corn steep liquor 5 g, CaCO_3_ 2 g, 6 ml/L trace elements solution (mg/mL): FeSO_4_⋅7H_2_O, 5.0; CuSO_4_⋅5H_2_O, 0.39; ZnSO_4_⋅7H_2_O, 0.44; MnSO_4_⋅H_2_O, 0.15; Na_2_MoO_4_⋅2H_2_O, 0.01; CoCl_2_⋅6H_2_O, 0.02; HCl, 50. In 1 L of distilled water) and A-3 M media (glucose 5 g, soluble starch 20 g, glycerol 20 g, cottonseed meal 15 g, yeast extract 3 g, in 1 L of distilled water, pH 7.2). Seeding cultures were prepared as previously described. 30 mL of fermentation medium was inoculated with 1.5 mL seeding culture and cultivated in 250 mL baffled flasks at 28 °C with 200 rpm for 7 days. All fermentation cultures from this study were freeze-dried and extracted with methanol. The organic phase was concentrated to 10% in vacuo.

Tentative identification of secondary metabolites in these extracts was achieved by LC-MS analysis using a Vanquish Horizon UHPLC system (Thermo Fisher Scientific) equipped with an Acquity Premier HSS T3 column, 2.1 × 150 mm, 1.8 μm (Waters) coupled to the ESI source of a timsTOF fleX mass spectrometer (Bruker Daltonics) as described previously^24^. Compass DataAnalysis 5.3 (Bruker Daltonics), GNPS, The Natural Products Atlas, and CAS SciFinder (American Chemical Society) were used for data analysis^41,42^.

### Co-cultivation experiments

Bacteria were cultivated separately in 100 mL flasks containing 10 mL V-22 seeding medium (soluble starch 10 g, glucose 5 g, NZ-case 3 g, yeast extract 2 g, Bacto tryptone 5 g, K_2_HPO_4_ 1 g, MgSO_4_⋅7H_2_O 5 g, CaCO_3_ 3 g, in 1 L of distilled water, pH 7.0) at 30 °C with 200 rpm for 2 days. Seeding cultures containing *S. bambergiensis* AC-800 or mutant strain (0.5 mL) and *Rhodococcus* sp. CML ISP2-1-52 (0.5 mL) were inoculated simultaneously into 250 mL baffled flasks containing 50 mL A-3 M fermentation medium and cultivated at 30 °C with 200 rpm for 7 days^26^. Mono-cultures were also inoculated using 0.5 mL seeding culture from each organism and cultivated as above.

## Supplementary Information

Below is the link to the electronic supplementary material.

Supplementary Material 1

## Data Availability

The datasets generated and/or analyzed during the current study are available in the NCBI repository, BioProject PRJNA1231356 (https://www.ncbi.nlm.nih.gov/bioproject/?term=PRJNA1231356).
