# Supplementary material for: Genome mining of Streptomyces bambergiensis AC-800 unravels the biosynthetic gene cluster for inhibitors of prolyl hydroxylase fibrostatins
Source: Sci Rep. 2025 Sep 1;15:32142. doi: 10.1038/s41598-025-17585-y (PMC12402327; doi:10.1038/s41598-025-17585-y)
Supplement: Supplementary file 1 — Supplementary Material 1 [file 41598_2025_17585_MOESM1_ESM.docx]

**Supplementary Information**

**Insights into the genome of** ***Streptomyces bambergiensis* Ac-800 and its secondary metabolites biosynthesis potential**

Jaime Felipe Guerrero-Garzón^1^, Olha Schneider^1^, Martin Zehl^2^, Inmaculada Tocino Marquez^1^, Christian Rückert-Reed^3,4^, Jörn Kalinowski^4^, Sergey B. Zotchev^1^*

^1^Department of Pharmaceutical Sciences, Division of Pharmacognosy, University of Vienna, 1090 Vienna, Austria; ^2^Department of Analytical Chemistry, Faculty of Chemistry, University of Vienna, 1090 Vienna, Austria; ^3^Medical School OWL, Bielefeld University, 33615 Bielefeld, Germany.

^4^Technology Platform Genomics, Center for Biotechnology (CeBiTec), Bielefeld University, 33615 Bielefeld, Germany.

**Table S1.** List of protospacers used for pCRISPR-cBEST system

| **Name** | **Sequence (5’-3’)** | **Target** |
| --- | --- | --- |
| KO_C1.6_Ga0065125_11963_sp1 | CGGTTGGTAGGATCGACGGC**ATCAGCCATGCGGTCATCGA**GTTTTAGAGCTAGAAATAGA | Ga0065125_11963, Region 1.6 |

* Protospacer in bold and light gray background flanked by overhangs compatible with pCRISPR-cBEST.

**Table S2.** List of primers used in this study

| **Name** | **Sequence (5’-3’)** | **Characteristics** |
| --- | --- | --- |
| KO_C1.6_Ga0065125_11963_Fw | GTCAAAGCTTGTACGGCGTAGGAGATCCAG | Screening of KO mutant, Ga0065125_11963 |
| KO_C1.6_Ga0065125_11963_Rv | GTCAGAATTCCTGAAATTCGCCGAAGCGAC | Screening of KO mutant, Ga0065125_11963 |

**Figure S1.** High resolution ESI-Qq-TOF mass spectrum of **Moenomycin A** in strain *S. bambergiensis* Ac-800 grown in 5288 (A) and high resolution MS/MS spectrum of its [M+H]^+^ ion (B).

**Figure S2.** High resolution ESI-Qq-TOF mass spectrum of **Nosokomycin B** in strain *S. bambergiensis* Ac-800 grown in 5288 (A) and high resolution MS/MS spectrum of its [M+H]^+^ ion (B).

**Figure S3.** High resolution ESI-Qq-TOF mass spectrum of **Moenomycin A_12_** in strain *S. bambergiensis* Ac-800 grown in NIIGEN (A) and high resolution MS/MS spectrum of its [M+H]^+^ ion (B).

**Figure S4.** High resolution ESI-Qq-TOF mass spectrum of **Desferrioxamine B** in strain *S. bambergiensis* Ac-800 grown in A-3M (A) and high resolution MS/MS spectrum of its [M+H]^+^ ion (B).

**Figure S5.** High resolution ESI-Qq-TOF mass spectrum of a known **hydroxamate sideropohore (CAS: 252325-60-3)** in strain *S. bambergiensis* Ac-800 grown in NIIGEN (A) and high resolution MS/MS spectrum of its [M+H]^+^ ion (B).

**Figure S6.** High resolution ESI-Qq-TOF mass spectrum of a known **hydroxamate sideropohore (CAS: 1884272-02-9)** in strain *S. bambergiensis* Ac-800 grown in NIIGEN (A) and high resolution MS/MS spectrum of its [M+H]^+^ ion (B).

**Figure S7.** High resolution ESI-Qq-TOF mass spectrum of **Terragine F (CAS: 2779605-10-4)** in strain *S. bambergiensis* Ac-800 grown in NIIGEN (A) and high resolution MS/MS spectrum of its [M+H]^+^ ion (B).

**Figure S8.** High resolution ESI-Qq-TOF mass spectrum of a **Desferrioxamine D_1_ (CAS: 5722-48-5)** in strain *S. bambergiensis* Ac-800 grown in NIIGEN (A) and high resolution MS/MS spectrum of its [M+H]^+^ ion (B).

**Figure S9.** High resolution ESI-Qq-TOF mass spectrum of **Gaburedin A** in strain *S. bambergiensis* Ac-800 grown in A-3M (A) and high resolution MS/MS spectrum of its [M+H]^+^ ion (B).

**Figure S10.** High resolution ESI-Qq-TOF mass spectrum of **Gaburedin B** in strain *S. bambergiensis* Ac-800 grown in A-3M (A) and high resolution MS/MS spectrum of its [M+H]^+^ ion (B).

**Figure S11.** High resolution ESI-Qq-TOF mass spectrum of **Gaburedin C** in strain *S. bambergiensis* Ac-800 grown in A-3M (A) and high resolution MS/MS spectrum of its [M+H]^+^ ion (B).

**Figure S12.** High resolution ESI-Qq-TOF mass spectrum of **Gaburedin D** in strain *S. bambergiensis* Ac-800 grown in A-3M (A) and high resolution MS/MS spectrum of its [M+H]^+^ ion (B).

**Figure S13.** High resolution ESI-Qq-TOF mass spectrum of a **polycyclic tetramate macrolactam** in strain *S. bambergiensis* Ac-800 grown in 5288 (A) and high resolution MS/MS spectrum of its [M+H]^+^ ion (B).

**Figure S14.** High resolution ESI-Qq-TOF mass spectrum of a **polycyclic tetramate macrolactam** in strain *S. bambergiensis* AC-800 grown in 5288 (A) and high resolution MS/MS spectrum of its [M+H]^+^ ion (B).

**Figure S15.** High resolution ESI-Qq-TOF mass spectrum of **Fibrostatin C/D/E** in strain *S. bambergiensis* AC-800 grown in co-culture with *Rhodococcus* sp. CML ISP2-1-52 in A-3M (A) and high resolution MS/MS spectrum of its [M+H]^+^ ion (B).

**Figure S16.** Extracted ion chromatograms (*m/z* 410.0904±0.0050) obtained by LC-MS in positive ion mode of the extracts from the monoculture of *Rhodococcus* sp. CML ISP2-1-52 (A) the monoculture of *S. bambergiensis* Ac-800 (B), the co-culture of the two strains (C), the monoculture of the *Ga0065125_11963* KO strain of *S. bambergiensis* Ac-800 (D) and the co-culture of the *Ga0065125_11963* KO strain of *S. bambergiensis* Ac-800 and *Rhodococcus* sp. CML ISP2-1-52 (E) all grown in A-3M.

**Figure S17.** Extracted ion chromatograms (*m/z* 410.0904±0.0050) obtained by LC-MS in positive ion mode of the extracts from *S. bambergiensis* Ac-800 grown in 5288 (A), from the *Ga0065125_11963* KO strain of *S. bambergiensis* Ac-800 grown in 5288 (B), from *S. bambergiensis* Ac-800 grown in PM4-1 (C), and from the *Ga0065125_11963* KO strain of *S. bambergiensis* Ac-800 grown in PM4-1 (D).
